# Crystal structure of ethyl 2-[(4-bromo­phen­yl)amino]-3,4-di­methyl­pent-3-enoate

**DOI:** 10.1107/S1600536814020832

**Published:** 2014-09-24

**Authors:** Julio Zukerman-Schpector, I. Caracelli, Hélio A. Stefani, Amna N. Khan, Edward R. T. Tiekink

**Affiliations:** a Departmento de Química, Universidade Federal de São Carlos, 13565-905 São Carlos, SP, Brazil; b Departmento de Física, Universidade Federal de São Carlos, 13565-905 São Carlos, SP, Brazil; c Departamento de Farmácia, Faculdade de Ciências Farmacêuticas, Universidade de São Paulo, São Paulo-SP, Brazil; d Department of Chemistry, University of Malaya, 50603 Kuala Lumpur, Malaysia

**Keywords:** crystal structure, hydrogen bonding, amine

## Abstract

In the title compound, C_15_H_20_BrNO_2_, there are two independent mol­ecules (*A* and *B*) comprising the asymmetric unit and these adopt very similar conformations. In *A*, the dihedral angle between the CO_2_ and MeC=CMe_2_ groups is 80.7 (3)°, and these make dihedral angles of 3.5 (3) and 84.09 (16)°, respectively, with the bromo­benzene ring. The equivalent dihedral angles for mol­ecule *B* are 78.4 (3), 2.1 (3) and 78.37 (12)°, respectively. The most prominent inter­actions in the crystal packing are amine-N—H⋯O(carbon­yl) hydrogen bonds between the two independent mol­ecules, resulting in non-centrosymmetric ten-membered {⋯OC_2_NH}_2_ synthons. Statistical disorder is noted for each of the terminal methyl groups of the ethyl residues.

## Related literature   

For background to the study into new and simpler synthetic routes for β,γ-unsaturated α-amino acid derivatives, see: Stefani *et al.* (2013 ▶). For the use of potassium organotri­fluoro­borate in synthesis, see: Caracelli *et al.* (2007 ▶).
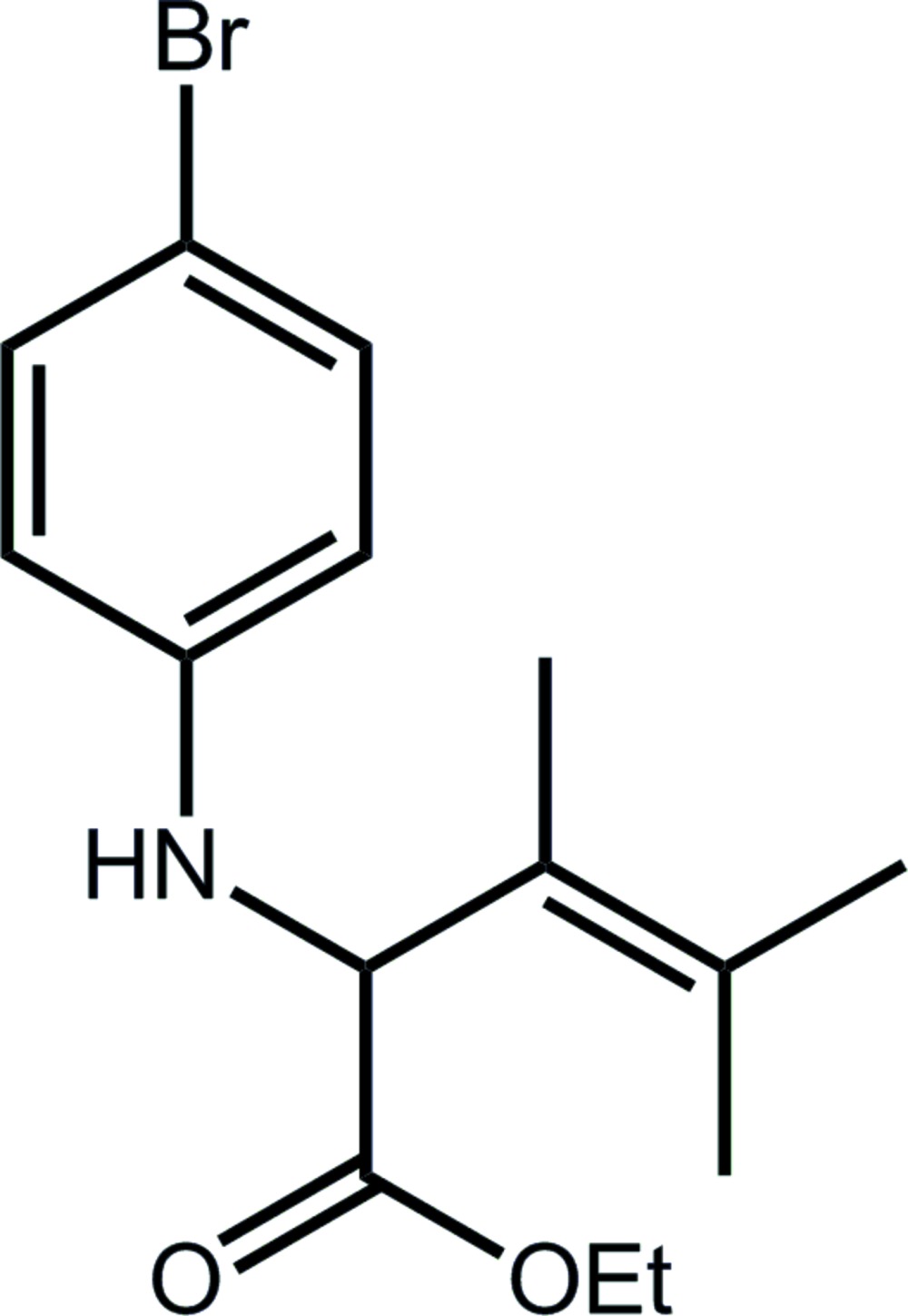


## Experimental   

### Crystal data  

C_15_H_20_BrNO_2_
*M* *_r_* = 326.22
Triclinic, 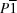

*a* = 11.8746 (6) Å
*b* = 12.2023 (5) Å
*c* = 13.7760 (6) Å
α = 97.557 (3)°
β = 110.520 (2)°
γ = 113.866 (2)°
*V* = 1620.20 (14) Å^3^
*Z* = 4
Mo *K*α radiation
μ = 2.54 mm^−1^
*T* = 290 K
0.50 × 0.34 × 0.28 mm

### Data collection  

Bruker Kappa APEXII CCD diffractometer
Absorption correction: multi-scan (*SADABS*; Sheldrick, 1996 ▶) *T*_min_ = 0.552, *T*_max_ = 0.745
18794 measured reflections
5927 independent reflections
4054 reflections with *I* > 2σ(*I*)
*R*_int_ = 0.026

### Refinement  

*R*[*F* ^2^ > 2σ(*F* ^2^)] = 0.045
*wR*(*F* ^2^) = 0.129
*S* = 1.03
5927 reflections
377 parameters
2 restraints
H atoms treated by a mixture of independent and constrained refinement
Δρ_max_ = 0.70 e Å^−3^
Δρ_min_ = −0.73 e Å^−3^

### 

Data collection: *APEX2* (Bruker, 2009 ▶); cell refinement: *SAINT* (Bruker, 2009 ▶); data reduction: *SAINT*; program(s) used to solve structure: *SIR97* (Altomare *et al.*, 1999 ▶); program(s) used to refine structure: *SHELXL97* (Sheldrick, 2008 ▶); molecular graphics: *ORTEP-3 for Windows* (Farrugia, 2012 ▶), *QMol* (Gans & Shalloway, 2001 ▶) and *DIAMOND* (Brandenburg, 2006 ▶); software used to prepare material for publication: *MarvinSketch* (Chemaxon, 2010 ▶) and *publCIF* (Westrip, 2010 ▶).

## Supplementary Material

Crystal structure: contains datablock(s) I. DOI: 10.1107/S1600536814020832/hg5408sup1.cif

Structure factors: contains datablock(s) I. DOI: 10.1107/S1600536814020832/hg5408Isup2.hkl

Supporting information file. DOI: 10.1107/S1600536814020832/hg5408Isup3.cml

**Fig. 1.**
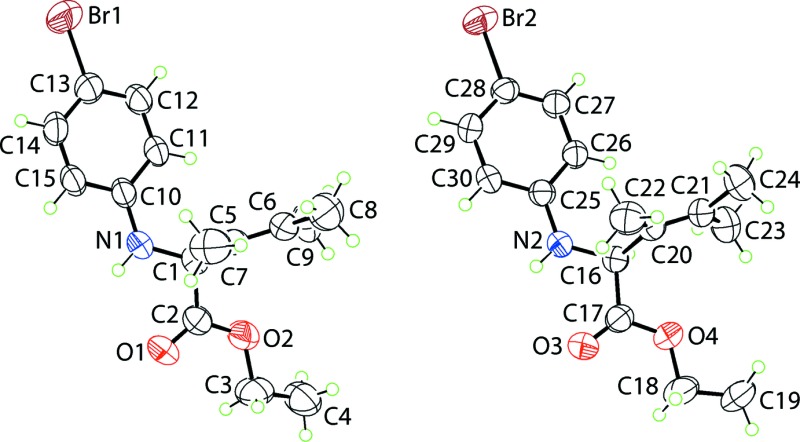
The molecular structures of the two independent molecules in the title compound showing the atom-labelling scheme and displacement ellipsoids at the 50% probability level.

. DOI: 10.1107/S1600536814020832/hg5408fig1.tif
The mol­ecular structures of the two independent mol­ecules in the title compound showing the atom-labelling scheme and displacement ellipsoids at the 50% probability level.

**Fig. 2.**
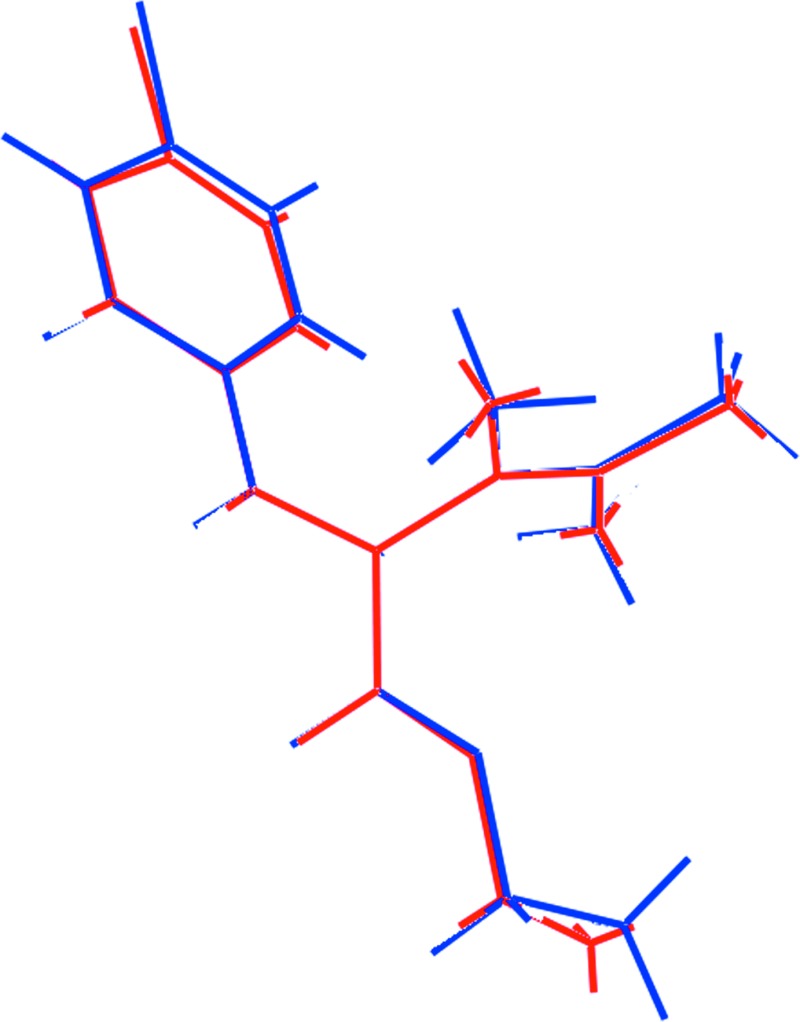
Overlay diagram of the two crystallographically independent molecules of the title compound. The N1- and N2-containing molecules are shown in red and blue, respectively.

. DOI: 10.1107/S1600536814020832/hg5408fig2.tif
Overlay diagram of the two crystallographically independent mol­ecules of the title compound. The N1- and N2-containing mol­ecules are shown in red and blue, respectively.

CCDC reference: 1024669

Additional supporting information:  crystallographic information; 3D view; checkCIF report

## Figures and Tables

**Table 1:** Hydrogen-bond geometry (Å, °)

*D*—H⋯*A*	*D*—H	H⋯*A*	*D*⋯*A*	*D*—H⋯*A*
N1—H1*N*⋯O3^i^	0.86	2.44	3.235 (6)	154
N2—H2*N*⋯O1^i^	0.86	2.37	3.153 (5)	153

Symmetry code: (i) 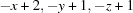.

## supplementary crystallographic information

### S5. Synthesis and crystallization

Ytterbium triflate (10 mol%) was added to a stirred solution of (*E*)-ethyl 2-(4-bromo­phenyl­imino)­acetate (0.5 mmol) in CH_2_Cl_2_ (5 mL). Potassium 3-methyl-2-buten-2-yltri­fluoro­borate (0.6 mmol) was then added and reaction mixture was stirred at room temperature until there was total consumption of the starting material. The reaction mixture was extracted with NaOH (0.5 N). The organic phase was dried using MgSO_4_, and the solvent was removed under reduced pressure. Suitable crystals were obtained by slow evaporation from its EtOAc solution.

^1^H NMR (CDCl_3_, 300 MHz): δ 7.18 (d, *J* = 8.7 Hz, 2*H*), 6.35 (d, *J* = 8.7 Hz, 2*H*), 4.86 (s,1*H*), 4.59 (bs, NH), 4.17-4.08 (m, 2*H*), 1.87 (s, 3*H*), 1.65 (s, 3*H*), 1.42 (s, 3*H*), 1.21 (t, *J* = 7.1 Hz, 3*H*) ppm. ^13^C NMR (CDCl_3_, 75 MHz) δ =171.9, 145.3, 131.8 (2C), 131.6, 123.6, 114.7 (2C), 109.2, 61.4, 57.7, 21.4, 20.5, 14.1, 12.6 ppm. HRMS: calcd. for C_15_H_20_BrNO_2_ [M + H]^+^ 325.0677; found: 325.0671.

### S6. Refinement

Carbon-bound H-atoms were placed in calculated positions (C—H = 0.93 to 0.98 Å) and were included in the refinement in the riding model approximation, with *U_iso_*(H) = 1.2–1.5*U_eq_*(C). The N—H H atoms were refined with N—H = 0.86±0.01 Å, and with *U_iso_*(H) = 1.2*U_eq_*(N). The terminal methyl group of each ethyl residue was found to be statistically disordered over two positions. These were refined so that equivalent pairs of atoms in each residue had the same anisotropic displacement parameters. Disorder in the C10–C15 benzene ring, manifested in a short average C—C bond length, *i.e*. 1.37 Å, could not be resolved.

### Crystal data

**Table:**

C_15_H_20_BrNO_2_	*Z* = 4
*M**_r_* = 326.22	*F*(000) = 672
Triclinic, *P*1	*D*_x_ = 1.337 Mg m^−^^3^
Hall symbol: -P 1	Mo *K*α radiation, λ = 0.71073 Å
*a* = 11.8746 (6) Å	Cell parameters from 6830 reflections
*b* = 12.2023 (5) Å	θ = 2.8–24.5°
*c* = 13.7760 (6) Å	µ = 2.54 mm^−^^1^
α = 97.557 (3)°	*T* = 290 K
β = 110.520 (2)°	Irregular, colourless
γ = 113.866 (2)°	0.50 × 0.34 × 0.28 mm
*V* = 1620.20 (14) Å^3^	

### Data collection

**Table:**

Bruker Kappa APEXII CCD diffractometer	5927 independent reflections
Radiation source: fine-focus sealed tube	4054 reflections with *I* > 2σ(*I*)
Graphite monochromator	*R*_int_ = 0.026
ω and φ scans	θ_max_ = 25.4°, θ_min_ = 1.7°
Absorption correction: multi-scan (*SADABS*; Sheldrick, 1996)	*h* = −13→14
*T*_min_ = 0.552, *T*_max_ = 0.745	*k* = −14→14
18794 measured reflections	*l* = −16→16

### Refinement

**Table:**

Refinement on *F*^2^	Primary atom site location: structure-invariant direct methods
Least-squares matrix: full	Secondary atom site location: difference Fourier map
*R*[*F*^2^ > 2σ(*F*^2^)] = 0.045	Hydrogen site location: inferred from neighbouring sites
*wR*(*F*^2^) = 0.129	H atoms treated by a mixture of independent and constrained refinement
*S* = 1.03	*w* = 1/[σ^2^(*F*_o_^2^) + (0.0596*P*)^2^ + 0.9501*P*] where *P* = (*F*_o_^2^ + 2*F*_c_^2^)/3
5927 reflections	(Δ/σ)_max_ < 0.001
377 parameters	Δρ_max_ = 0.70 e Å^−^^3^
2 restraints	Δρ_min_ = −0.73 e Å^−^^3^

### Special details

**Table:**

Geometry. All s.u.'s (except the s.u. in the dihedral angle between two l.s. planes) are estimated using the full covariance matrix. The cell s.u.'s are taken into account individually in the estimation of s.u.'s in distances, angles and torsion angles; correlations between s.u.'s in cell parameters are only used when they are defined by crystal symmetry. An approximate (isotropic) treatment of cell s.u.'s is used for estimating s.u.'s involving l.s. planes.
Refinement. Refinement of *F*^2^ against ALL reflections. The weighted *R*-factor *wR* and goodness of fit *S* are based on *F*^2^, conventional *R*-factors *R* are based on *F*, with *F* set to zero for negative *F*^2^. The threshold expression of *F*^2^ > 2σ(*F*^2^) is used only for calculating *R*-factors(gt) *etc*. and is not relevant to the choice of reflections for refinement. *R*-factors based on *F*^2^ are statistically about twice as large as those based on *F*, and *R*- factors based on ALL data will be even larger.

### Fractional atomic coordinates and isotropic or equivalent isotropic displacement parameters (Å^2^)

**Table:**

	*x*	*y*	*z*	*U*_iso_*/*U*_eq_	Occ. (<1)
Br1	0.40749 (8)	0.86878 (7)	0.59824 (4)	0.1400 (3)	
O1	1.0407 (3)	0.8284 (3)	1.1303 (2)	0.0801 (8)	
O2	1.0548 (2)	0.9633 (3)	1.2646 (2)	0.0763 (7)	
N1	0.8516 (4)	0.8629 (4)	0.9787 (3)	0.0780 (10)	
H1N	0.889 (4)	0.819 (3)	0.966 (3)	0.094*	
C1	0.8827 (3)	0.9110 (3)	1.0912 (3)	0.0566 (8)	
H1	0.9102	1.0012	1.1093	0.068*	
C2	1.0017 (3)	0.8954 (3)	1.1617 (3)	0.0613 (9)	
C3	1.1615 (4)	0.9495 (4)	1.3454 (3)	0.0851 (12)	0.50
H3A	1.2353	0.9647	1.3248	0.102*	0.50
H3B	1.1241	0.8643	1.3495	0.102*	0.50
C4	1.2124 (17)	1.0377 (15)	1.4481 (16)	0.095 (5)	0.50
H4A	1.2291	1.1189	1.4397	0.143*	0.50
H4B	1.2968	1.0441	1.4979	0.143*	0.50
H4C	1.1460	1.0101	1.4763	0.143*	0.50
C3'	1.1615 (4)	0.9495 (4)	1.3454 (3)	0.0851 (12)	0.50
H3C	1.2506	1.0009	1.3483	0.102*	0.50
H3D	1.1412	0.8620	1.3300	0.102*	0.50
C4'	1.157 (2)	0.998 (2)	1.4551 (17)	0.191 (14)	0.50
H4D	1.1003	1.0376	1.4427	0.286*	0.50
H4E	1.2485	1.0579	1.5098	0.286*	0.50
H4F	1.1195	0.9276	1.4797	0.286*	0.50
C5	0.7633 (3)	0.8432 (3)	1.1185 (3)	0.0526 (8)	
C6	0.7384 (3)	0.9023 (3)	1.1894 (3)	0.0576 (8)	
C7	0.6809 (4)	0.7015 (3)	1.0618 (3)	0.0844 (12)	
H7A	0.6281	0.6623	1.0985	0.127*	
H7B	0.7424	0.6686	1.0637	0.127*	
H7C	0.6200	0.6839	0.9871	0.127*	
C8	0.6271 (4)	0.8335 (5)	1.2224 (4)	0.0909 (13)	
H8A	0.5672	0.7488	1.1726	0.136*	
H8B	0.5751	0.8770	1.2204	0.136*	
H8C	0.6684	0.8310	1.2951	0.136*	
C9	0.8196 (4)	1.0425 (4)	1.2481 (3)	0.0801 (11)	
H9A	0.8852	1.0590	1.3207	0.120*	
H9B	0.7583	1.0736	1.2520	0.120*	
H9C	0.8671	1.0844	1.2090	0.120*	
C10	0.7498 (4)	0.8649 (3)	0.8936 (3)	0.0547 (8)	
C11	0.6902 (4)	0.9391 (3)	0.9089 (3)	0.0610 (9)	
H11	0.7187	0.9884	0.9792	0.073*	
C12	0.5895 (4)	0.9404 (3)	0.8214 (3)	0.0646 (9)	
H12	0.5513	0.9912	0.8324	0.077*	
C13	0.5462 (4)	0.8675 (4)	0.7189 (3)	0.0696 (10)	
C14	0.6019 (4)	0.7914 (4)	0.7010 (3)	0.0709 (10)	
H14	0.5709	0.7407	0.6307	0.085*	
C15	0.7030 (4)	0.7917 (3)	0.7878 (3)	0.0614 (9)	
H15	0.7414	0.7414	0.7756	0.074*	
Br2	0.49175 (4)	0.41287 (4)	−0.39558 (3)	0.07843 (18)	
O3	1.0151 (3)	0.2650 (2)	0.13441 (19)	0.0672 (6)	
O4	1.0539 (3)	0.4148 (3)	0.27261 (18)	0.0744 (7)	
N2	0.8714 (3)	0.3402 (3)	−0.0138 (2)	0.0595 (7)	
H2N	0.888 (4)	0.280 (2)	−0.031 (3)	0.071*	
C16	0.9097 (3)	0.3973 (3)	0.0989 (2)	0.0481 (7)	
H16	0.9642	0.4892	0.1181	0.058*	
C17	0.9983 (3)	0.3501 (3)	0.1685 (3)	0.0524 (8)	
C18	1.1336 (4)	0.3766 (4)	0.3526 (3)	0.0819 (12)	0.50
H18A	1.2144	0.3865	0.3438	0.098*	0.50
H18B	1.0790	0.2890	0.3457	0.098*	0.50
C19	1.1732 (15)	0.4617 (17)	0.4594 (11)	0.126 (6)	0.50
H19A	1.2409	0.5454	0.4705	0.189*	0.50
H19B	1.2107	0.4314	0.5166	0.189*	0.50
H19C	1.0936	0.4636	0.4605	0.189*	0.50
C18'	1.1336 (4)	0.3766 (4)	0.3526 (3)	0.0819 (12)	0.50
H18C	1.2309	0.4325	0.3777	0.098*	0.50
H18D	1.1129	0.2913	0.3197	0.098*	0.50
C19'	1.0989 (17)	0.3819 (14)	0.4482 (13)	0.117 (5)	0.50
H19D	1.1248	0.4675	0.4831	0.175*	0.50
H19E	1.1482	0.3524	0.4997	0.175*	0.50
H19F	1.0019	0.3292	0.4225	0.175*	0.50
C20	0.7878 (3)	0.3681 (3)	0.1252 (2)	0.0484 (7)	
C21	0.7868 (3)	0.4541 (3)	0.1947 (3)	0.0550 (8)	
C22	0.6735 (4)	0.2335 (3)	0.0699 (3)	0.0700 (10)	
H22A	0.6291	0.2086	0.1156	0.105*	
H22B	0.7108	0.1793	0.0576	0.105*	
H22C	0.6078	0.2266	0.0011	0.105*	
C23	0.9035 (4)	0.5876 (4)	0.2554 (3)	0.0807 (11)	
H23A	0.9629	0.5913	0.3257	0.121*	
H23B	0.8670	0.6433	0.2649	0.121*	
H23C	0.9547	0.6130	0.2143	0.121*	
C24	0.6692 (4)	0.4254 (4)	0.2237 (3)	0.0864 (12)	
H24A	0.5903	0.3479	0.1715	0.130*	
H24B	0.6474	0.4930	0.2227	0.130*	
H24C	0.6950	0.4169	0.2955	0.130*	
C25	0.7873 (3)	0.3598 (3)	−0.0999 (2)	0.0471 (7)	
C26	0.7547 (3)	0.4562 (3)	−0.0847 (2)	0.0520 (8)	
H26	0.7915	0.5107	−0.0146	0.062*	
C27	0.6683 (3)	0.4717 (3)	−0.1726 (2)	0.0532 (8)	
H27	0.6465	0.5360	−0.1614	0.064*	
C28	0.6147 (3)	0.3936 (3)	−0.2755 (2)	0.0498 (7)	
C29	0.6476 (3)	0.2985 (3)	−0.2934 (3)	0.0560 (8)	
H29	0.6119	0.2460	−0.3640	0.067*	
C30	0.7330 (3)	0.2825 (3)	−0.2061 (3)	0.0542 (8)	
H30	0.7550	0.2186	−0.2183	0.065*	

### Atomic displacement parameters (Å^2^)

**Table:**

	*U*^11^	*U*^22^	*U*^33^	*U*^12^	*U*^13^	*U*^23^
Br1	0.1954 (7)	0.2199 (7)	0.0665 (3)	0.1728 (6)	0.0380 (3)	0.0441 (4)
O1	0.0827 (18)	0.110 (2)	0.0766 (17)	0.0718 (17)	0.0375 (15)	0.0241 (15)
O2	0.0632 (15)	0.1006 (19)	0.0620 (16)	0.0502 (14)	0.0184 (13)	0.0091 (14)
N1	0.093 (2)	0.121 (3)	0.0558 (18)	0.083 (2)	0.0354 (17)	0.0231 (18)
C1	0.061 (2)	0.070 (2)	0.0554 (19)	0.0412 (18)	0.0312 (17)	0.0200 (16)
C2	0.052 (2)	0.077 (2)	0.062 (2)	0.0338 (18)	0.0294 (17)	0.0218 (19)
C3	0.057 (2)	0.098 (3)	0.077 (3)	0.040 (2)	0.008 (2)	0.014 (2)
C4	0.082 (8)	0.103 (7)	0.086 (10)	0.055 (6)	0.020 (6)	−0.001 (6)
C3'	0.057 (2)	0.098 (3)	0.077 (3)	0.040 (2)	0.008 (2)	0.014 (2)
C4'	0.22 (3)	0.36 (4)	0.056 (8)	0.21 (3)	0.033 (14)	0.049 (15)
C5	0.0490 (18)	0.0557 (18)	0.0530 (18)	0.0303 (15)	0.0173 (15)	0.0164 (15)
C6	0.054 (2)	0.072 (2)	0.0556 (19)	0.0354 (17)	0.0268 (16)	0.0246 (17)
C7	0.079 (3)	0.063 (2)	0.089 (3)	0.033 (2)	0.020 (2)	0.013 (2)
C8	0.064 (3)	0.128 (4)	0.091 (3)	0.041 (3)	0.048 (2)	0.047 (3)
C9	0.100 (3)	0.075 (2)	0.080 (3)	0.048 (2)	0.053 (2)	0.017 (2)
C10	0.070 (2)	0.0635 (19)	0.0572 (19)	0.0426 (17)	0.0411 (18)	0.0254 (16)
C11	0.080 (2)	0.073 (2)	0.0539 (19)	0.052 (2)	0.0379 (18)	0.0191 (17)
C12	0.089 (3)	0.076 (2)	0.066 (2)	0.060 (2)	0.046 (2)	0.0313 (19)
C13	0.097 (3)	0.088 (3)	0.059 (2)	0.065 (2)	0.042 (2)	0.037 (2)
C14	0.106 (3)	0.084 (2)	0.052 (2)	0.065 (2)	0.043 (2)	0.0235 (18)
C15	0.087 (3)	0.071 (2)	0.063 (2)	0.056 (2)	0.048 (2)	0.0292 (18)
Br2	0.0834 (3)	0.0954 (3)	0.0568 (2)	0.0548 (2)	0.01649 (19)	0.0287 (2)
O3	0.0790 (17)	0.0841 (16)	0.0601 (14)	0.0583 (14)	0.0297 (13)	0.0270 (13)
O4	0.0727 (16)	0.1072 (19)	0.0448 (13)	0.0582 (15)	0.0137 (12)	0.0153 (13)
N2	0.0765 (19)	0.0811 (19)	0.0456 (15)	0.0580 (17)	0.0288 (14)	0.0222 (14)
C16	0.0514 (18)	0.0561 (17)	0.0445 (17)	0.0303 (15)	0.0236 (14)	0.0185 (14)
C17	0.0427 (18)	0.069 (2)	0.0476 (18)	0.0255 (16)	0.0226 (15)	0.0216 (16)
C18	0.065 (2)	0.120 (3)	0.057 (2)	0.051 (2)	0.0153 (19)	0.030 (2)
C19	0.123 (13)	0.23 (2)	0.051 (6)	0.124 (13)	0.023 (8)	0.043 (11)
C18'	0.065 (2)	0.120 (3)	0.057 (2)	0.051 (2)	0.0153 (19)	0.030 (2)
C19'	0.144 (15)	0.153 (14)	0.063 (7)	0.079 (10)	0.045 (10)	0.038 (9)
C20	0.0478 (18)	0.0601 (18)	0.0423 (16)	0.0306 (15)	0.0189 (14)	0.0197 (14)
C21	0.057 (2)	0.069 (2)	0.0473 (17)	0.0355 (17)	0.0266 (16)	0.0196 (16)
C22	0.057 (2)	0.067 (2)	0.076 (2)	0.0245 (18)	0.0259 (19)	0.0204 (19)
C23	0.092 (3)	0.075 (2)	0.073 (3)	0.045 (2)	0.035 (2)	0.008 (2)
C24	0.083 (3)	0.121 (3)	0.081 (3)	0.061 (3)	0.052 (2)	0.029 (3)
C25	0.0487 (18)	0.0571 (17)	0.0464 (17)	0.0295 (15)	0.0261 (14)	0.0219 (14)
C26	0.065 (2)	0.0538 (17)	0.0441 (17)	0.0350 (16)	0.0241 (15)	0.0148 (14)
C27	0.068 (2)	0.0529 (17)	0.0511 (18)	0.0365 (16)	0.0285 (16)	0.0221 (15)
C28	0.0492 (18)	0.0592 (18)	0.0470 (17)	0.0289 (15)	0.0225 (14)	0.0232 (15)
C29	0.062 (2)	0.070 (2)	0.0398 (17)	0.0363 (17)	0.0232 (15)	0.0134 (15)
C30	0.062 (2)	0.0629 (19)	0.0507 (19)	0.0393 (17)	0.0292 (16)	0.0152 (15)

### Geometric parameters (Å, º)

**Table:**

Br1—C13	1.894 (4)	Br2—C28	1.900 (3)
O1—C2	1.194 (4)	O3—C17	1.201 (4)
O2—C2	1.318 (4)	O4—C17	1.317 (4)
O2—C3	1.451 (4)	O4—C18	1.445 (4)
N1—C10	1.369 (4)	N2—C25	1.378 (4)
N1—C1	1.438 (4)	N2—C16	1.437 (4)
N1—H1N	0.858 (10)	N2—H2N	0.856 (10)
C1—C2	1.509 (5)	C16—C17	1.513 (4)
C1—C5	1.531 (5)	C16—C20	1.530 (4)
C1—H1	0.9800	C16—H16	0.9800
C3—C4	1.413 (17)	C18—C19	1.477 (16)
C3—H3A	0.9700	C18—H18A	0.9700
C3—H3B	0.9700	C18—H18B	0.9700
C4—H4A	0.9600	C19—H19A	0.9600
C4—H4B	0.9600	C19—H19B	0.9600
C4—H4C	0.9600	C19—H19C	0.9600
C4'—H4D	0.9600	C19'—H19D	0.9600
C4'—H4E	0.9600	C19'—H19E	0.9600
C4'—H4F	0.9600	C19'—H19F	0.9600
C5—C6	1.323 (5)	C20—C21	1.331 (4)
C5—C7	1.515 (5)	C20—C22	1.504 (4)
C6—C9	1.505 (5)	C21—C24	1.505 (5)
C6—C8	1.511 (5)	C21—C23	1.508 (5)
C7—H7A	0.9600	C22—H22A	0.9600
C7—H7B	0.9600	C22—H22B	0.9600
C7—H7C	0.9600	C22—H22C	0.9600
C8—H8A	0.9600	C23—H23A	0.9600
C8—H8B	0.9600	C23—H23B	0.9600
C8—H8C	0.9600	C23—H23C	0.9600
C9—H9A	0.9600	C24—H24A	0.9600
C9—H9B	0.9600	C24—H24B	0.9600
C9—H9C	0.9600	C24—H24C	0.9600
C10—C15	1.386 (4)	C25—C30	1.390 (4)
C10—C11	1.392 (4)	C25—C26	1.393 (4)
C11—C12	1.376 (5)	C26—C27	1.379 (4)
C11—H11	0.9300	C26—H26	0.9300
C12—C13	1.356 (5)	C27—C28	1.358 (4)
C12—H12	0.9300	C27—H27	0.9300
C13—C14	1.382 (5)	C28—C29	1.385 (4)
C14—C15	1.364 (5)	C29—C30	1.371 (4)
C14—H14	0.9300	C29—H29	0.9300
C15—H15	0.9300	C30—H30	0.9300
			
C2—O2—C3	117.8 (3)	C17—O4—C18	118.3 (3)
C10—N1—C1	123.0 (3)	C25—N2—C16	123.7 (2)
C10—N1—H1N	120 (3)	C25—N2—H2N	116 (2)
C1—N1—H1N	116 (3)	C16—N2—H2N	119 (2)
N1—C1—C2	108.2 (3)	N2—C16—C17	108.2 (2)
N1—C1—C5	114.0 (3)	N2—C16—C20	114.1 (3)
C2—C1—C5	107.8 (3)	C17—C16—C20	108.4 (2)
N1—C1—H1	108.9	N2—C16—H16	108.7
C2—C1—H1	108.9	C17—C16—H16	108.7
C5—C1—H1	108.9	C20—C16—H16	108.7
O1—C2—O2	124.1 (3)	O3—C17—O4	124.5 (3)
O1—C2—C1	125.4 (3)	O3—C17—C16	125.2 (3)
O2—C2—C1	110.5 (3)	O4—C17—C16	110.3 (3)
O2—C3—C4	108.7 (8)	O4—C18—C19	104.7 (6)
O2—C3—H3A	109.9	O4—C18—H18A	110.8
C4—C3—H3A	109.9	C19—C18—H18A	110.8
O2—C3—H3B	109.9	O4—C18—H18B	110.8
C4—C3—H3B	109.9	C19—C18—H18B	110.8
H3A—C3—H3B	108.3	H18A—C18—H18B	108.9
H4D—C4'—H4E	109.5	H19D—C19'—H19E	109.5
H4D—C4'—H4F	109.5	H19D—C19'—H19F	109.5
H4E—C4'—H4F	109.5	H19E—C19'—H19F	109.5
C6—C5—C7	123.1 (3)	C21—C20—C22	123.5 (3)
C6—C5—C1	122.9 (3)	C21—C20—C16	122.2 (3)
C7—C5—C1	114.0 (3)	C22—C20—C16	114.2 (3)
C5—C6—C9	124.2 (3)	C20—C21—C24	122.7 (3)
C5—C6—C8	122.6 (3)	C20—C21—C23	124.3 (3)
C9—C6—C8	113.1 (3)	C24—C21—C23	113.0 (3)
C5—C7—H7A	109.5	C20—C22—H22A	109.5
C5—C7—H7B	109.5	C20—C22—H22B	109.5
H7A—C7—H7B	109.5	H22A—C22—H22B	109.5
C5—C7—H7C	109.5	C20—C22—H22C	109.5
H7A—C7—H7C	109.5	H22A—C22—H22C	109.5
H7B—C7—H7C	109.5	H22B—C22—H22C	109.5
C6—C8—H8A	109.5	C21—C23—H23A	109.5
C6—C8—H8B	109.5	C21—C23—H23B	109.5
H8A—C8—H8B	109.5	H23A—C23—H23B	109.5
C6—C8—H8C	109.5	C21—C23—H23C	109.5
H8A—C8—H8C	109.5	H23A—C23—H23C	109.5
H8B—C8—H8C	109.5	H23B—C23—H23C	109.5
C6—C9—H9A	109.5	C21—C24—H24A	109.5
C6—C9—H9B	109.5	C21—C24—H24B	109.5
H9A—C9—H9B	109.5	H24A—C24—H24B	109.5
C6—C9—H9C	109.5	C21—C24—H24C	109.5
H9A—C9—H9C	109.5	H24A—C24—H24C	109.5
H9B—C9—H9C	109.5	H24B—C24—H24C	109.5
N1—C10—C15	120.0 (3)	N2—C25—C30	119.9 (3)
N1—C10—C11	122.3 (3)	N2—C25—C26	122.2 (3)
C15—C10—C11	117.7 (3)	C30—C25—C26	117.9 (3)
C12—C11—C10	120.8 (3)	C27—C26—C25	120.6 (3)
C12—C11—H11	119.6	C27—C26—H26	119.7
C10—C11—H11	119.6	C25—C26—H26	119.7
C13—C12—C11	119.8 (3)	C28—C27—C26	120.3 (3)
C13—C12—H12	120.1	C28—C27—H27	119.9
C11—C12—H12	120.1	C26—C27—H27	119.9
C12—C13—C14	120.8 (3)	C27—C28—C29	120.4 (3)
C12—C13—Br1	120.0 (3)	C27—C28—Br2	120.0 (2)
C14—C13—Br1	119.2 (3)	C29—C28—Br2	119.6 (2)
C15—C14—C13	119.2 (3)	C30—C29—C28	119.5 (3)
C15—C14—H14	120.4	C30—C29—H29	120.3
C13—C14—H14	120.4	C28—C29—H29	120.3
C14—C15—C10	121.6 (3)	C29—C30—C25	121.2 (3)
C14—C15—H15	119.2	C29—C30—H30	119.4
C10—C15—H15	119.2	C25—C30—H30	119.4
			
C10—N1—C1—C2	−178.9 (3)	C25—N2—C16—C17	178.2 (3)
C10—N1—C1—C5	61.1 (5)	C25—N2—C16—C20	57.4 (4)
C3—O2—C2—O1	−3.9 (6)	C18—O4—C17—O3	−4.1 (5)
C3—O2—C2—C1	173.9 (3)	C18—O4—C17—C16	175.2 (3)
N1—C1—C2—O1	−16.7 (5)	N2—C16—C17—O3	−10.7 (4)
C5—C1—C2—O1	107.1 (4)	C20—C16—C17—O3	113.5 (3)
N1—C1—C2—O2	165.4 (3)	N2—C16—C17—O4	170.0 (3)
C5—C1—C2—O2	−70.8 (4)	C20—C16—C17—O4	−65.7 (3)
C2—O2—C3—C4	174.9 (9)	C17—O4—C18—C19	−176.6 (7)
N1—C1—C5—C6	−144.0 (3)	N2—C16—C20—C21	−141.6 (3)
C2—C1—C5—C6	95.8 (4)	C17—C16—C20—C21	97.8 (3)
N1—C1—C5—C7	39.1 (4)	N2—C16—C20—C22	41.0 (4)
C2—C1—C5—C7	−81.2 (3)	C17—C16—C20—C22	−79.7 (3)
C7—C5—C6—C9	179.1 (3)	C22—C20—C21—C24	−1.0 (5)
C1—C5—C6—C9	2.5 (5)	C16—C20—C21—C24	−178.2 (3)
C7—C5—C6—C8	1.4 (5)	C22—C20—C21—C23	176.7 (3)
C1—C5—C6—C8	−175.2 (3)	C16—C20—C21—C23	−0.5 (5)
C1—N1—C10—C15	−164.7 (3)	C16—N2—C25—C30	−167.1 (3)
C1—N1—C10—C11	15.6 (6)	C16—N2—C25—C26	13.5 (5)
N1—C10—C11—C12	178.9 (3)	N2—C25—C26—C27	−178.9 (3)
C15—C10—C11—C12	−0.9 (5)	C30—C25—C26—C27	1.6 (5)
C10—C11—C12—C13	0.9 (6)	C25—C26—C27—C28	−0.6 (5)
C11—C12—C13—C14	0.0 (6)	C26—C27—C28—C29	−0.7 (5)
C11—C12—C13—Br1	179.9 (3)	C26—C27—C28—Br2	178.2 (2)
C12—C13—C14—C15	−0.9 (6)	C27—C28—C29—C30	1.0 (5)
Br1—C13—C14—C15	179.2 (3)	Br2—C28—C29—C30	−178.0 (2)
C13—C14—C15—C10	0.9 (6)	C28—C29—C30—C25	0.1 (5)
N1—C10—C15—C14	−179.9 (4)	N2—C25—C30—C29	179.2 (3)
C11—C10—C15—C14	−0.1 (5)	C26—C25—C30—C29	−1.4 (5)

### Hydrogen-bond geometry (Å, º)

**Table:**

*D*—H···*A*	*D*—H	H···*A*	*D*···*A*	*D*—H···*A*
N1—H1*N*···O3^i^	0.86	2.44	3.235 (6)	154
N2—H2*N*···O1^i^	0.86	2.37	3.153 (5)	153

Symmetry code: (i) −*x*+2, −*y*+1, −*z*+1.

## References

[bb1] Altomare, A., Burla, M. C., Camalli, M., Cascarano, G. L., Giacovazzo, C., Guagliardi, A., Moliterni, A. G. G., Polidori, G. & Spagna, R. (1999). *J. Appl. Cryst.* **32**, 115–119.

[bb2] Brandenburg, K. (2006). *DIAMOND* Crystal Impact GbR, Bonn, Germany.

[bb3] Bruker (2009). *APEX2* and *SAINT* Bruker AXS Inc., Madison, Wisconsin, USA.

[bb4] Caracelli, I., Stefani, H. A., Vieira, A. S., Machado, M. M. P. & Zukerman-Schpector, J. (2007). *Z. Kristallogr. New Cryst. Struct.* **222**, 345–347.

[bb5] Chemaxon (2010). *Marvinsketch.* http://www.chemaxon.com

[bb6] Farrugia, L. J. (2012). *J. Appl. Cryst.* **45**, 849–854.

[bb7] Gans, J. D. & Shalloway, D. (2001). *J. Mol. Graph. Model.* **19**, 557–559. 10.1016/s1093-3263(01)00090-0 11552684

[bb8] Sheldrick, G. M. (1996). *SADABS* University of Göttingen, Germany.

[bb9] Sheldrick, G. M. (2008). *Acta Cryst.* A**64**, 112–122. 10.1107/S0108767307043930 18156677

[bb10] Stefani, H. A., Khan, A. N., Manarin, F., Vendramini, P. H. & Eberlin, M. N. (2013). *Tetrahedron Lett.* **54**, 6204–6207.

[bb11] Westrip, S. P. (2010). *J. Appl. Cryst.* **43**, 920–925.

