# Supplementary figures and images for: Crystal structure of ethyl 2-[(4-bromo­phen­yl)amino]-3,4-di­methyl­pent-3-enoate
Source: Acta Crystallogr Sect E Struct Rep Online. 2014 Sep 24;70(Pt 10):o1122–3. doi: 10.1107/S1600536814020832 (PMC4257164; doi:10.1107/S1600536814020832)

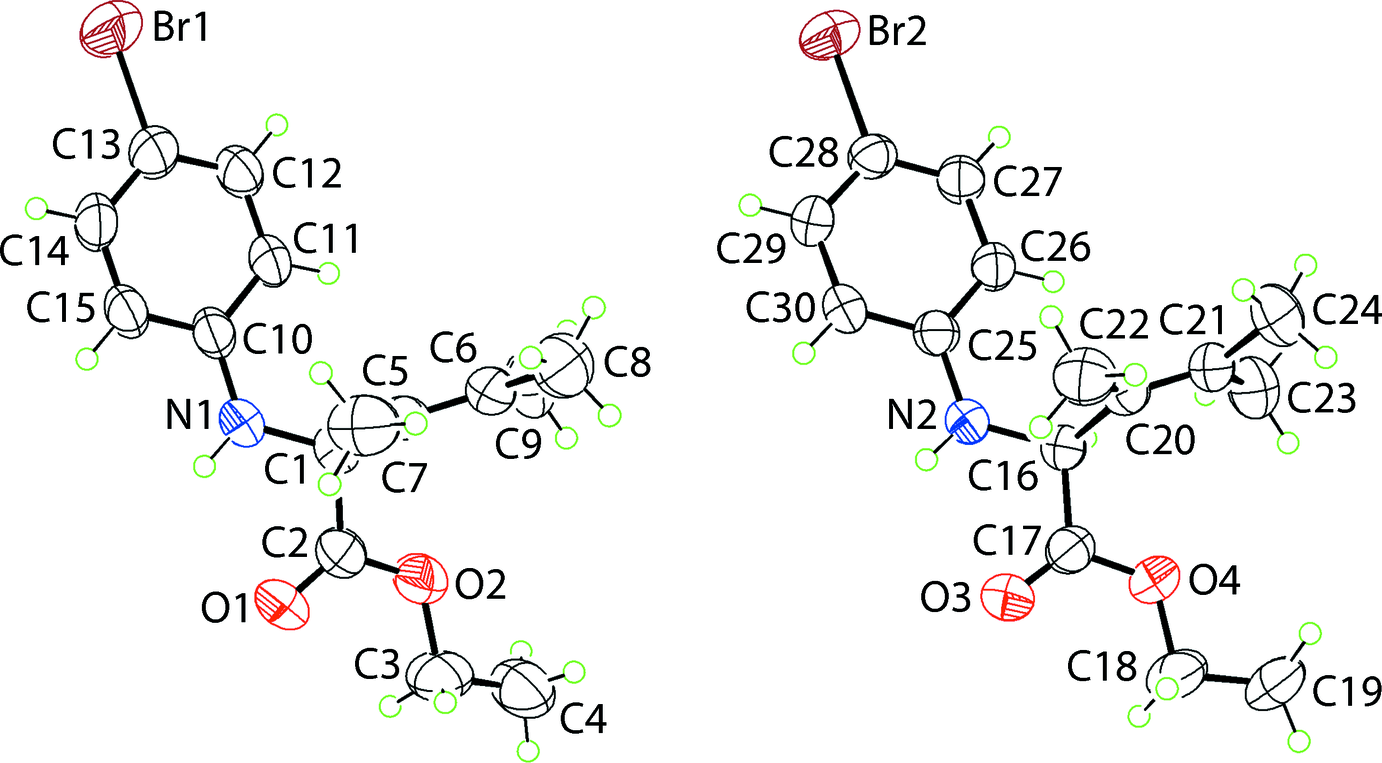

Supplement: Supplementary file 4 [file e-70-o1122-fig1.tif]

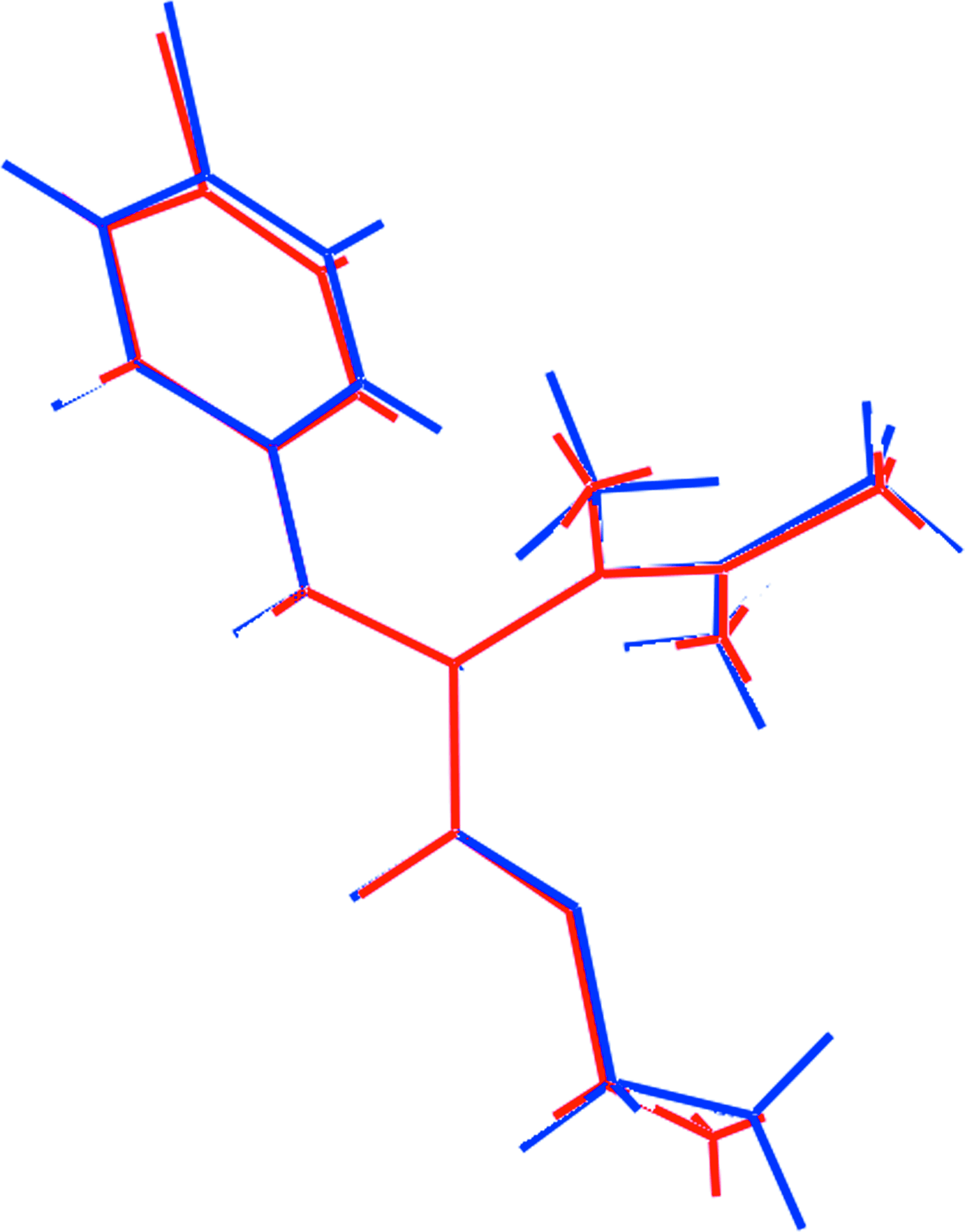

Supplement: Supplementary file 5 [file e-70-o1122-fig2.tif]
